# Supplementary material for: Properties of “Stable” Mosquito Cytochrome P450 Enzymes
Source: Insects. 2025 Feb 8;16(2):184. doi: 10.3390/insects16020184 (PMC11855896; doi:10.3390/insects16020184)
Supplement: Supplementary file 1 [file insects-16-00184-s001.zip › GTzotzos_SI/SI3.pdf.pdf]

Unculled "labile" CYPs (BUSCA output)

CYP ID	GOids	GOterms	Score	Signal Peptide	Mitochondrion Transit Peptide	Transmembrane Alpha Helix	1st TMH	2nd TMH	3rd TMH	4th TMH
**AAEL001292**	GO:0012505	C:endomembrane system	0.94			2-24;317-345;476-494	2-24	317-345	476-494	
**AAEL001312**	GO:0012505	C:endomembrane system	0.95			2-24;317-345;476-494	2-24	317-345	476-494	
**AAEL001320**	GO:0012505	C:endomembrane system	0.95			3-23;315-343;474-492	3-23	315-343	474-492	
**AAEL001807**	GO:0012505	C:endomembrane system	0.95			2-21;314-342;473-491	2-21	314-342	473-491	
**AAEL001960**	GO:0031966	C:mitochondrial membrane	0.69		1-29	315-343;474-492	315-343	474-492		
**AAEL002005**	GO:0012505	C:endomembrane system	0.89			238-266;397-415	238-266	397-415		
**AAEL002031**	GO:0031966	C:mitochondrial membrane	0.76		1-19	313-339;470-488	313-339	470-488		
**AAEL002085**	GO:0012505	C:endomembrane system	0.95			2-20;298-326;453-471	2-20	298-326	453-471	
**AAEL002633**	GO:0012505	C:endomembrane system	0.94			3-23;319-347;478-496	3-23	319-347	478-496	
**AAEL002638**	GO:0012505	C:endomembrane system	0.94			3-23;319-347;478-496	3-23	319-347	478-496	
**AAEL003380**	GO:0012505	C:endomembrane system	0.95			11-39;315-343;471-489	11-39	315-343	471-489	
**AAEL003399**	GO:0012505	C:endomembrane system	0.94	1-20		294-322;449-467	294-322	449-467		
**AAEL003748**	GO:0012505	C:endomembrane system	0.94			2-23;313-341;472-490	2-23	313-341	472-490	
**AAEL006784**	GO:0012505	C:endomembrane system	0.95			3-24;324-352;482-500	3-24	324-352	482-500	
**AAEL006795**	GO:0012505	C:endomembrane system	0.94			3-24;322-350;481-499	3-24	322-350	481-499	
**AAEL006805**	GO:0012505	C:endomembrane system	0.94			3-25;321-349;480-498	3-25	321-349	480-498	
**AAEL006815**	GO:0012505	C:endomembrane system	0.95			3-23;325-353;483-501	3-23	325-353	483-501	
**AAEL006827**	GO:0031966	C:mitochondrial membrane	0.63		1-25	301-329;458-476	301-329	458-476		
**AAEL006984**	GO:0012505	C:endomembrane system	0.95			2-23;286-314;445-464	2-23	286-314	445-464	
**AAEL006989**	GO:0012505	C:endomembrane system	0.95			2-22;284-312;444-462	2-22	284-312	444-462	
**AAEL006992**	GO:0012505	C:endomembrane system	0.94			2-22;284-312;444-462	2-22	284-312	444-462	
**AAEL007010**	GO:0012505	C:endomembrane system	0.94			2-24;284-312;443-461	2-24	284-312	443-461	
**AAEL007024**	GO:0012505	C:endomembrane system	0.94			2-23;284-312;443-461	2-23	284-312	443-461	
**AAEL007795**	GO:0012505	C:endomembrane system	0.93			17-49;197-208;319-347;474-492	17-49	197-208	319-347	474-492
**AAEL007798**	GO:0012505	C:endomembrane system	0.95			2-20;290-318;443-461	2-20	290-318	443-461	
**AAEL007807**	GO:0012505	C:endomembrane system	0.94	1-22		292-320;447-465	292-320	;447-465		
**AAEL007812**	GO:0012505	C:endomembrane system	0.95			2-21;293-321;449-467	2-21	293-321	449-467	
**AAEL007816**	GO:0012505	C:endomembrane system	0.95			2-23;294-322;449-467	2-23	294-322	449-467	
**AAEL007830**	GO:0012505	C:endomembrane system	0.95			3-28;306-334;461-479	3-28	306-334	461-479	
**AAEL008017**	GO:0012505	C:endomembrane system	0.93			10-41;195-206;338-366;491-510	10-41	195-206	338-366	491-510
**AAEL008018**	GO:0012505	C:endomembrane system	0.95			23-54;339-367;493-511	23-54	339-367	493-511	
**AAEL008638**	GO:0031090	C:organelle membrane	0.83			320-347;482-501	320-347	482-501		
**AAEL008889**	GO:0012505	C:endomembrane system	0.95			2-21;298-326;453-471	2-21	298-326	453-471	
**AAEL009018**	GO:0012505	C:endomembrane system	0.95			2-20;296-324;447-465	2-20	296-324	447-465	
**AAEL009117**	GO:0012505	C:endomembrane system	0.95			3-24;285-313;440-458	3-24	285-313	440-458	
**AAEL009121**	GO:0012505	C:endomembrane system	0.94			2-20;292-320;446-464	2-20	292-320	446-464	
**AAEL009122**	GO:0012505	C:endomembrane system	0.93			2-20;290-318;443-461	2-20	290-318	443-461	
**AAEL009124**	GO:0012505	C:endomembrane system	0.94			2-20;286-314;440-458	2-20	286-314	440-458	
**AAEL009125**	GO:0012505	C:endomembrane system	0.95			2-23;285-313;440-458	2-23	285-313	440-458	
**AAEL009126**	GO:0012505	C:endomembrane system	0.93			2-20;291-319;445-463	2-20	291-319	445-463	
**AAEL009127**	GO:0012505	C:endomembrane system	0.94			3-24;291-319;446-464	3-24	291-319	446-464	
**AAEL009128**	GO:0012505	C:endomembrane system	0.93	1-17		283-311;437-456	283-311	437-456		
**AAEL009133**	GO:0012505	C:endomembrane system	0.93			2-20;290-318;444-462	2-20	290-318	444-462	
**AAEL009137**	GO:0012505	C:endomembrane system	0.94			2-20;290-318;444-462	2-20	290-318	444-462	
**AAEL009138**	GO:0012505	C:endomembrane system	0.94			2-20;290-318;444-462	2-20	290-318	444-462	
**AAEL009591**	GO:0012505	C:endomembrane system	0.89			29-57;114-125;354-382;512-531	29-57	114-125	354-382	512-531
**AAEL009656**	GO:0012505	C:endomembrane system	0.95			2-21;300-328;450-469	2-21	300-328	450-469	
**AAEL010151**	GO:0012505	C:endomembrane system	0.93			2-20;290-318;444-462	2-20	290-318	444-462	
**AAEL010158**	GO:0012505	C:endomembrane system	0.94			2-21;292-320;446-464	2-21	292-320	446-464	
**AAEL011761**	GO:0012505	C:endomembrane system	0.9			3-23;299-327;454-472	3-23	299-327	454-472	
**AAEL012266**	GO:0012505	C:endomembrane system	0.95			3-24;304-332;457-475	3-24	304-332	457-475	
**AAEL012765**	GO:0012505	C:endomembrane system	0.95			3-23;291-319;446-464	3-23	291-319	446-464	
**AAEL013798**	GO:0012505	C:endomembrane system	0.94			2-20;299-327;454-472	2-20	299-327	454-472	
**AAEL014603**	GO:0012505	C:endomembrane system	0.93			3-23;324-352;483-501	3-23	324-352	483-501	
**AAEL014605**	GO:0012505	C:endomembrane system	0.94			3-24;327-355;486-504	3-24;	327-355	486-504	
**AAEL014609**	GO:0012505	C:endomembrane system	0.94			3-23;323-351;482-500	3-23	323-351	482-500	
**AAEL014610**	GO:0012505	C:endomembrane system	0.94			3-23;324-352;483-501	3-23	324-352	483-501	
**AAEL014614**	GO:0012505	C:endomembrane system	0.95			3-24;327-355;486-504	3-24	327-355	486-504	
**AAEL014615**	GO:0012505	C:endomembrane system	0.94			3-24;318-346;477-495	3-24	318-346	477-495	
**AAEL014617**	GO:0012505	C:endomembrane system	0.95			3-23;322-350;481-499	3-23	322-350	481-499	
**AAEL014619**	GO:0012505	C:endomembrane system	0.94			3-24;322-350;480-498	3-24	322-350	480-498	
**AAEL014678**	GO:0012505	C:endomembrane system	0.95			2-23;299-327;451-469	2-23	299-327	451-469	
**AAEL014684**	GO:0012505	C:endomembrane system	0.95			2-24;299-327;451-469	2-24	299-327	451-469	

**AAEL014893**	GO:0012505	C:endomembrane system	0.93			3-23;297-325;452-470	3-23	297-325	452-470	
**AAEL017297**	GO:0012505	C:endomembrane system	0.94			3-24;285-313;440-458	3-24	285-313	440-458	
**AAEL017366**	GO:0012505	C:endomembrane system	0.94			3-23;323-351;482-500	3-23	323-351	482-500	
**AAEL019504**	GO:0012505	C:endomembrane system	0.95			3-23;324-352;483-501	3-23	324-352	483-501	
**AAEL019603**	GO:0012505	C:endomembrane system	0.95			3-23;324-352;482-500	3-23	324-352	482-500	
**AAEL020553**	GO:0012505	C:endomembrane system	0.94			3-24;298-326;453-471	3-24	298-326	453-471	
**AAEL022568**	GO:0012505	C:endomembrane system	0.94			2-23;294-322;449-467	2-23	294-322	449-467	
**AAEL025214**	GO:0012505	C:endomembrane system	0.84	1-19		274-302;428-446	274-302	428-446		
**AAEL025530**	GO:0012505	C:endomembrane system	0.94			2-24;317-345;476-494	2-24	317-345	476-494	
**AAEL028635**	GO:0012505	C:endomembrane system	0.94			2-24;326-354;485-503	2-24	326-354	485-503	
**AGAP000088**	GO:0012505	C:endomembrane system	0.95			2-19;302-330;457-475	2-19	302-330	457-475	
**AGAP000192**	GO:0012505	C:endomembrane system	0.94			16-45;332-358;486-505	16-45	332-358	486-505	
**AGAP000193**	GO:0012505	C:endomembrane system	0.87			23-53;206-217;339-367;496-514	23-53	206-217	339-367	496-514
**AGAP000194**	GO:0012505	C:endomembrane system	0.95			10-41;348-376;501-520	10-41	348-376	501-520	
**AGAP001861**	GO:0012505	C:endomembrane system	0.95			2-21;298-326;453-471	2-21	298-326	453-471	
**AGAP001864**	GO:0012505	C:endomembrane system	0.94			3-26;304-332;459-477	3-26	304-332	459-477	
**AGAP002206**	GO:0012505	C:endomembrane system	0.95			3-27;289-317;443-461	3-27	289-317	443-461	
**AGAP002210**	GO:0012505	C:endomembrane system	0.95			2-20;291-319;446-464	2-20	291-319	446-464	
**AGAP002418**	GO:0012505	C:endomembrane system	0.94			2-24;299-327;454-472	2-24	299-327	454-472	
**AGAP002419**	GO:0012505	C:endomembrane system	0.94			2-23;300-328;455-473	2-23	300-328	455-473	
**AGAP002865**	GO:0012505	C:endomembrane system	0.94			3-24;301-329;456-474	3-24	301-329	456-474	
**AGAP002866**	GO:0012505	C:endomembrane system	0.93			2-24;300-328;455-473	2-24	300-328	455-473	
**AGAP002867**	GO:0012505	C:endomembrane system	0.9			3-24;298-326;453-471	3-24	298-326	453-471	
**AGAP002868**	GO:0012505	C:endomembrane system	0.94			2-24;301-329;455-474	2-24	301-329	455-474	
**AGAP002869**	GO:0012505	C:endomembrane system	0.93			2-25;299-327;454-472	2-25	299-327	454-472	
**AGAP003608**	GO:0012505	C:endomembrane system	0.92			8-35;290-318;442-461	8-35	290-318	442-461	
**AGAP005774**	GO:0031090	C:organelle membrane	0.9			17-47;221-232;332-360;495-514	17-47	221-232	332-360	495-514
**AGAP006047**	GO:0012505	C:endomembrane system	0.94			2-23;298-326;454-472	2-23	298-326	454-472	
**AGAP008019**	GO:0031966	C:mitochondrial membrane	0.7		1-30	312-340;471-489	312-340	471-489		
**AGAP008020**	GO:0031966	C:mitochondrial membrane	0.59		1-28	311-339;470-488	311-339	470-488		
**AGAP008022**	GO:0031090	C:organelle membrane	0.89			311-339;471-489	311-339	471-489		
**AGAP008206**	GO:0012505	C:endomembrane system	0.94			2-21;292-320;446-464	2-21	292-320	446-464	
**AGAP008207**	GO:0012505	C:endomembrane system	0.94			2-29;302-330;457-475	2-29	302-330	457-475	
**AGAP008208**	GO:0012505	C:endomembrane system	0.95			3-24;294-322;449-467	3-24	294-322	449-467	
**AGAP008209**	GO:0012505	C:endomembrane system	0.93			3-26;289-318;445-463	3-26	289-318	445-463	
**AGAP008212**	GO:0012505	C:endomembrane system	0.93			3-24;291-319;446-464	3-24	291-319	446-464	
**AGAP008213**	GO:0012505	C:endomembrane system	0.94			2-23;290-318;445-463	2-23	290-318	445-463	
**AGAP008214**	GO:0012505	C:endomembrane system	0.95			3-24;290-318;445-463	3-24	290-318	445-463	
**AGAP008356**	GO:0012505	C:endomembrane system	0.94	1-24		291-319;446-464	291-319	446-464		
**AGAP008358**	GO:0012505	C:endomembrane system	0.95			2-20;299-327;454-472	2-20	299-327	454-472	
**AGAP008552**	GO:0012505	C:endomembrane system	0.92			16-48;198-209;327-355;482-500	16-48	198-209	327-355	482-500
**AGAP009240**	GO:0012505	C:endomembrane system	0.95			4-30;312-340;466-484	4-30	312-340	466-484	
**AGAP009246**	GO:0012505	C:endomembrane system	0.95			3-25;304-332;458-476	3-25	304-332	458-476	
**AGAP010414**	GO:0012505	C:endomembrane system	0.94			11-39;324-352;478-496	11-39	324-352	478-496	
**AGAP012291**	GO:0012505	C:endomembrane system	0.94			3-24;334-362;494-512	3-24	334-362	494-512	
**AGAP012292**	GO:0012505	C:endomembrane system	0.94			3-24;325-353;484-502	3-24	325-353	484-502	
**AGAP012294**	GO:0012505	C:endomembrane system	0.94			3-23;322-350;482-500	3-23	322-350	482-500	
**AGAP012295**	GO:0012505	C:endomembrane system	0.95			3-23;320-348;477-495	3-23	320-348	477-495	
**AGAP012296**	GO:0012505	C:endomembrane system	0.94			3-24;328-356;487-505	3-24	328-356	487-505	
**AGAP012957**	GO:0012505	C:endomembrane system	0.95			3-24;300-328;455-473	3-24	300-328	455-473	
**AGAP013224**	GO:0012505	C:endomembrane system	0.95			4-27;308-336;454-473	4-27	308-336	454-473	
**AGAP013241**	GO:0012505	C:endomembrane system	0.95			3-23;302-330;457-475	3-23	302-330	457-475	
**AGAP013305**	GO:0012505	C:endomembrane system	0.94			2-24;304-332;459-477	2-24	304-332	459-477	
**AGAP013490**	GO:0012505	C:endomembrane system	0.95			2-20;302-330;457-475	2-20	302-330	457-475	
**AGAP028019**	GO:0012505	C:endomembrane system	0.94	1-28		298-326;453-471	298-326	453-471		
**CPIJ000067**	GO:0012505	C:endomembrane system	0.86			20-45;216-227;326-354;489-508	20-45	216-227	;326-354	489-508
**CPIJ001757**	GO:0012505	C:endomembrane system	0.94	1-15		298-326;453-471	298-326	453-471		
**CPIJ001758**	GO:0012505	C:endomembrane system	0.93	1-17		299-327;454-472	299-327	454-472		
**CPIJ001759**	GO:0012505	C:endomembrane system	0.89	1-17		172-183;299-327	172-183	299-327		
**CPIJ001886**	GO:0012505	C:endomembrane system	0.95			2-22;298-326;453-471	2-22	298-326	453-471	
**CPIJ002535**	GO:0012505	C:endomembrane system	0.94			2-24;285-313;447-465	2-24	285-313	447-465	
**CPIJ002536**	GO:0012505	C:endomembrane system	0.94			2-22;284-312;444-462	2-22	284-312	444-462	
**CPIJ002537**	GO:0012505	C:endomembrane system	0.95			2-22;284-312;444-462	2-22	284-312	444-462	
**CPIJ002538**	GO:0012505	C:endomembrane system	0.95			3-24;287-315;446-464	3-24	287-315	446-464	
**CPIJ003082**	GO:0012505	C:endomembrane system	0.95			3-23;320-348;479-497	3-23	320-348	479-497	
**CPIJ003361**	GO:0012505	C:endomembrane system	0.94			2-22;284-312;438-456	2-22	284-312	438-456	

**CPIJ003375**	GO:0012505	C:endomembrane system	0.95			2-23;282-310;436-454	2-23	282-310	436-454	
**CPIJ003376**	GO:0012505	C:endomembrane system	0.94			2-21;280-308;434-452	2-21	280-308	434-452	
**CPIJ003377**	GO:0012505	C:endomembrane system	0.95			3-23;282-310;436-454	3-23	282-310	436-454	
**CPIJ003378**	GO:0012505	C:endomembrane system	0.95			2-23;282-310;435-455	2-23	282-310	435-455	
**CPIJ003389**	GO:0012505	C:endomembrane system	0.95			2-23;282-310;436-454	2-23	282-310	436-454	
**CPIJ004410**	GO:0012505	C:endomembrane system	0.91	1-22		250-278;403-422	250-278	403-422		
**CPIJ004411**	GO:0012505	C:endomembrane system	0.93			2-22;283-311;436-455	2-22	283-311	436-455	
**CPIJ005332**	GO:0012505	C:endomembrane system	0.94			3-23;318-346;477-495	3-23	318-346	477-495	
**CPIJ005900**	GO:0012505	C:endomembrane system	0.94			2-21;291-319;436-454	2-21	291-319	436-454	
**CPIJ005952**	GO:0012505	C:endomembrane system	0.95			2-24;290-318;445-463	2-24	290-318	445-463	
**CPIJ005953**	GO:0012505	C:endomembrane system	0.94			3-24;297-325;452-470	3-24	297-325	452-470	
**CPIJ005955**	GO:0012505	C:endomembrane system	0.95			2-24;305-333;460-478	2-24	305-333	460-478	
**CPIJ005956**	GO:0012505	C:endomembrane system	0.93			4-28;297-326;451-469	4-28	297-326	451-469	
**CPIJ006721**	GO:0012505	C:endomembrane system	0.95			2-21;299-327;457-475	2-21	299-327	457-475	
**CPIJ007089**	GO:0012505	C:endomembrane system	0.71			287-315;443-461	287-315	443-461		
**CPIJ007090**	GO:0012505	C:endomembrane system	0.95			2-23;292-320;446-465	2-23	292-320	446-465	
**CPIJ007091**	GO:0012505	C:endomembrane system	0.95			2-22;292-320;446-464	2-22	292-320	446-464	
**CPIJ007092**	GO:0012505	C:endomembrane system	0.95			2-21;291-319;444-463	2-21	291-319	444-463	
**CPIJ007093**	GO:0012505	C:endomembrane system	0.95			2-22;292-320;446-464	2-22	292-320	446-464	
**CPIJ007095**	GO:0012505	C:endomembrane system	0.95			2-20;291-319;445-463	2-20	291-319	445-463	
**CPIJ007188**	GO:0012505	C:endomembrane system	0.95			2-21;293-321;448-466	2-21	293-321	448-466	
**CPIJ008566**	GO:0012505	C:endomembrane system	0.94			2-22;281-309;435-453	2-22	281-309	435-453	
**CPIJ008936**	GO:0012505	C:endomembrane system	0.94			3-25;305-333;460-478	3-25	305-333	460-478	
**CPIJ008937**	GO:0012505	C:endomembrane system	0.95			3-26;305-333;460-478	3-26	305-333	460-478	
**CPIJ009085**	GO:0012505	C:endomembrane system	0.95			2-22;282-310;442-460	2-22	282-310	442-460	
**CPIJ009468**	GO:0012505	C:endomembrane system	0.95			2-22;294-322;447-466	2-22	294-322	447-466	
**CPIJ009476**	GO:0012505	C:endomembrane system	0.93			4-27;301-329;456-474	4-27	301-329	456-474	
**CPIJ009477**	GO:0012505	C:endomembrane system	0.9			247-275;402-420	247-275	402-420		
**CPIJ009478**	GO:0012505	C:endomembrane system	0.94	1-22		293-321;447-466	293-321	447-466		
**CPIJ010075**	GO:0012505	C:endomembrane system	0.95			3-26;305-333;460-478	3-26	305-333	460-478	
**CPIJ010175**	GO:0012505	C:endomembrane system	0.94			2-23;322-350;480-498	2-23	322-350	480-498	
**CPIJ010225**	GO:0031966	C:mitochondrial membrane	0.73		1-34	315-343;473-491	315-343	473-491		
**CPIJ010227**	GO:0031966	C:mitochondrial membrane	0.72		1-34	315-343;473-491	315-343	473-491		
**CPIJ010228**	GO:0031966	C:mitochondrial membrane	0.62		1-24	308-336;466-484	308-336	466-484		
**CPIJ010229**	GO:0031090	C:organelle membrane	0.87			2-23;312-340;471-489	2-23	312-340	471-489	
**CPIJ010230**	GO:0031090	C:organelle membrane	0.86			301-328;461-479	301-328	461-479		
**CPIJ010231**	GO:0031966	C:mitochondrial membrane	0.69		1-24	310-338;468-486	310-338	468-486		
**CPIJ010536**	GO:0012505	C:endomembrane system	0.95			3-25;326-354;485-503	3-25	326-354	485-503	
**CPIJ010537**	GO:0012505	C:endomembrane system	0.94			3-25;327-355;486-504	3-25	327-355	486-504	
**CPIJ010538**	GO:0012505	C:endomembrane system	0.94			2-24;323-351;482-500	2-24	323-351	482-500	
**CPIJ010539**	GO:0012505	C:endomembrane system	0.94			3-24;326-354;485-503	3-24	326-354	485-503	
**CPIJ010540**	GO:0012505	C:endomembrane system	0.72			288-316;447-465	288-316	447-465		
**CPIJ010541**	GO:0012505	C:endomembrane system	0.94			3-25;328-356;487-505	3-25	328-356	487-505	
**CPIJ010542**	GO:0012505	C:endomembrane system	0.94			2-23;324-352;483-501	2-23	324-352	483-501	
**CPIJ010543**	GO:0012505	C:endomembrane system	0.95			3-22;322-350;480-498	3-22	322-350	480-498	
**CPIJ010544**	GO:0012505	C:endomembrane system	0.94			3-23;321-349;480-498	3-23	321-349	480-498	
**CPIJ010545**	GO:0012505	C:endomembrane system	0.94			3-24;317-345;476-494	3-24	317-345	476-494	
**CPIJ010546**	GO:0012505	C:endomembrane system	0.94			3-23;321-349;480-498	3-23	321-349	480-498	
**CPIJ010547**	GO:0012505	C:endomembrane system	0.95			3-23;324-352;483-501	3-23	324-352	483-501	
**CPIJ010548**	GO:0012505	C:endomembrane system	0.94			3-23;322-350;481-499	3-23	322-350	481-499	
**CPIJ010858**	GO:0012505	C:endomembrane system	0.95			2-23;297-325;450-468	2-23	297-325	450-468	
**CPIJ011127**	GO:0012505	C:endomembrane system	0.94	1-18		295-323;451-469	295-323	451-469		
**CPIJ011129**	GO:0012505	C:endomembrane system	0.93			2-20;288-316;442-460	2-20	288-316	442-460	
**CPIJ011836**	GO:0012505	C:endomembrane system	0.92	1-21		146-157;258-287;412-430	146-157	258-287	412-430	
**CPIJ014940**	GO:0012505	C:endomembrane system	0.9	1-21		72-83;278-307;429-447	72-83	278-307	429-447	
**CPIJ014941**	GO:0012505	C:endomembrane system	0.93			2-17;285-314;436-454	2-17	285-314	436-454	
**CPIJ014942**	GO:0012505	C:endomembrane system	0.93	1-21		285-313;434-454	285-313	434-454		
**CPIJ015223**	GO:0012505	C:endomembrane system	0.95			2-22;299-327;451-469	2-22	299-327	451-469	
**CPIJ015428**	GO:0012505	C:endomembrane system	0.94			2-22;286-314;440-458	2-22	286-314	440-458	
**CPIJ015681**	GO:0012505	C:endomembrane system	0.92			2-21;298-327;457-481	2-21	298-327	457-481	
**CPIJ016846**	GO:0012505	C:endomembrane system	0.93			3-24;292-320;447-465	3-24	292-320	447-465	
**CPIJ016848**	GO:0012505	C:endomembrane system	0.94			3-24;291-319;446-464	3-24	291-319	446-464	
**CPIJ016850**	GO:0012505	C:endomembrane system	0.95			3-29;298-326;453-471	3-29	298-326	453-471	
**CPIJ016852**	GO:0012505	C:endomembrane system	0.94			2-21;291-319;445-463	2-21	291-319	445-463	
**CPIJ016854**	GO:0012505	C:endomembrane system	0.94			2-20;284-312;418-437	2-20	284-312	418-437	
**CPIJ016855**	GO:0012505	C:endomembrane system	0.94			2-21;291-319;445-463	2-21	291-319	445-463	

**CPIJ016856**	GO:0012505	C:endomembrane system	0.93			2-20;290-318;443-462	2-20	290-318	443-462	
**CPIJ017014**	GO:0012505	C:endomembrane system	0.94			2-22;284-312;443-462	2-22	284-312	443-462	
**CPIJ017351**	GO:0012505	C:endomembrane system	0.95			13-43;332-360;486-504	13-43	332-360	486-504	
**CPIJ017462**	GO:0012505	C:endomembrane system	0.92			2-20;278-306;432-450	2-20	278-306	432-450	
**CPIJ018854**	GO:0012505	C:endomembrane system	0.95			13-43;332-360;486-504	13-43	332-360	486-504	
**CPIJ018943**	GO:0012505	C:endomembrane system	0.95			5-36;321-349;476-494	5-36	321-349	476-494	
**CPIJ018944**	GO:0012505	C:endomembrane system	0.94			10-44;327-355;481-499	10-44	327-355	481-499	
**CPIJ019395**	GO:0005886	C:plasma membrane	0.67			239-267;394-412;448-467	239-267	394-412	448-467	
**CPIJ019586**	GO:0012505	C:endomembrane system	0.95			2-22;264-292;417-436	2-22	264-292	417-436	
**CPIJ019587**	GO:0012505	C:endomembrane system	0.93			2-21;282-310;435-453	2-21	282-310	435-453	
**CPIJ019673**	GO:0012505	C:endomembrane system	0.94			2-22;284-312;443-462	2-22	284-312	443-462	
**CPIJ019700**	GO:0012505	C:endomembrane system	0.93			3-25;292-320;447-465	3-25	292-320	447-465	
**CPIJ019702**	GO:0012505	C:endomembrane system	0.94			3-24;291-319;446-464	3-24	291-319	446-464	
**CPIJ019704**	GO:0012505	C:endomembrane system	0.93			2-21;253-278;404-422	2-21	253-278	404-422	
**CPIJ019751**	GO:0012505	C:endomembrane system	0.94			3-23;284-312;444-462	3-23	284-312	444-462	
**CPIJ020229**	GO:0012505	C:endomembrane system	0.72			134-145;259-287;413-432	134-145	259-287	413-432	
**FBgn0000473**	GO:0012505	C:endomembrane system	0.93	1-16		296-324;452-470	296-324	452-470		
**FBgn0011576**	GO:0012505	C:endomembrane system	0.95			2-22;298-326;451-469	2-22	298-326	451-469	
**FBgn0013771**	GO:0012505	C:endomembrane system	0.94			3-23;295-323;451-469	3-23	295-323	451-469	
**FBgn0013772**	GO:0012505	C:endomembrane system	0.94			3-25;297-325;453-471	3-25	297-325	453-471	
**FBgn0014469**	GO:0012505	C:endomembrane system	0.96			2-23;294-322;446-463	2-23	294-322	446-463	
**FBgn0015032**	GO:0012505	C:endomembrane system	0.94			6-21;23-47;329-357;483-501	6-21	23-47	329-357	483-501
**FBgn0015034**	GO:0012505	C:endomembrane system	0.96			2-22;293-322;446-463	2-22	293-322	446-463	
**FBgn0015035**	GO:0012505	C:endomembrane system	0.95	1-19		293-321;443-461	293-321	443-461		
**FBgn0015038**	GO:0012505	C:endomembrane system	0.95			2-23;291-319;451-469	2-23	291-319	451-469	
**FBgn0015039**	GO:0012505	C:endomembrane system	0.95			2-22;291-319;451-469	2-22	291-319	451-469	
**FBgn0015040**	GO:0012505	C:endomembrane system	0.95			2-24;306-334;466-484	2-24	306-334	466-484	
**FBgn0015714**	GO:0012505	C:endomembrane system	0.94			2-20;291-319;446-464	2-20	291-319	446-464	
**FBgn0025454**	GO:0012505	C:endomembrane system	0.93			3-23;299-327;460-478	3-23	299-327	460-478	
**FBgn0030615**	GO:0012505	C:endomembrane system	0.95			2-24;293-322;438-455	2-24	293-322	438-455	
**FBgn0033065**	GO:0012505	C:endomembrane system	0.93	1-18		291-319;451-469	291-319	451-469		
**FBgn0033292**	GO:0012505	C:endomembrane system	0.95			2-23;292-320;447-465	2-23	292-320	447-465	
**FBgn0033302**	GO:0012505	C:endomembrane system	0.95			2-23;300-328;456-474	2-23	300-328	456-474	
**FBgn0033304**	GO:0012505	C:endomembrane system	0.95			2-22;282-310;437-455	2-22	282-310	437-455	
**FBgn0033524**	GO:0031966	C:mitochondrial membrane	0.6		1-14	375-403;537-555	375-403	537-555		
**FBgn0033775**	GO:0012505	C:endomembrane system	0.94			3-24;306-334;463-482	3-24	306-334	463-482	
**FBgn0033978**	GO:0012505	C:endomembrane system	0.94			2-21;292-320;447-465	2-21	292-320	447-465	
**FBgn0033980**	GO:0012505	C:endomembrane system	0.94			2-22;292-320;447-465	2-22	292-320	447-465	
**FBgn0034053**	GO:0012505	C:endomembrane system	0.94			8-36;298-327;452-470	8-36	298-327	452-470	
**FBgn0034387**	GO:0031090	C:organelle membrane	0.8			318-346;480-499	318-346	480-499		
**FBgn0036806**	GO:0031090	C:organelle membrane	0.88			310-338;472-490	310-338	472-490		
**FBgn0038037**	GO:0012505	C:endomembrane system	0.93	1-22		303-331;461-479	303-331	461-479		
**FBgn0038680**	GO:0031090	C:organelle membrane	0.86			320-348;484-502	320-348	484-502		
**FBgn0038681**	GO:0031966	C:mitochondrial membrane	0.72		1-32	319-347;483-501	319-347	483-501		
**FBgn0039519**	GO:0012505	C:endomembrane system	0.92			2-25;297-325;453-471	2-25	297-325	453-471	
**FBgn0288232**	GO:0012505	C:endomembrane system	0.94			3-23;295-323;451-469	3-23	295-323	451-469	

Unculled “stable” CYPs (BUSCA output)

CYP ID	GOids	GOterms	Score	Signal Peptide	Mitochondrion Transit Peptide	Transmembrane Alpha Helix	1st TMH	2nd TMH	3rd TMH	4th TMH	5th TMH
**AAEL019660**	GO:0005886	C:plasma membrane	0,96			310-334	310-334				
**CPIJ005899**	GO:0005886	C:plasma membrane	0,73			114-139;422-450	114-139	422-450			
**AAEL005700**	GO:0031090	C:organelle membrane	0,7			8-36;306-334;459-477	8-36	306-334	459-477		
**AAEL014594**	GO:0031090	C:organelle membrane	0,91			375-403;532-550	375-403	532-550			
**AGAP000284**	GO:0031090	C:organelle membrane	0,94			288-316;433-452	288-316	433-452			
**AGAP005992**	GO:0031090	C:organelle membrane	0,87			309-337;465-483	309-337	465-483			
**CPIJ006322**	GO:0031090	C:organelle membrane	0,81			199-227;389-408	199-227	389-408			
**CPIJ015953**	GO:0031090	C:organelle membrane	0,91			6-32;305-333	6-32	305-333			
**FBgn0000449**	GO:0031090	C:organelle membrane	0,81			283-311;435-454	283-311	435-454			
**FBgn0003312**	GO:0031090	C:organelle membrane	0,89			10-39;328-356;468-486	10-39	328-356	468-486		
**FBgn0037817**	GO:0031090	C:organelle membrane	0,88			4-21;310-338;470-488	4-21	310-338	470-488		
**AGAP008018**	GO:0031966	C:mitochondrial membrane	0,76		1-22	310-337;469-487	310-337	469-487			
**CPIJ008980**	GO:0031966	C:mitochondrial membrane	0,74		1-62	367-394;523-541	367-394	523-541			
**FBgn0033753**	GO:0031966	C:mitochondrial membrane	0,74		1-34	347-374;502-521	347-374	502-521			
**FBgn0050489**	GO:0031966	C:mitochondrial membrane	0,62		1-30	311-339;468-486	311-339	468-486			
**FBgn0053503**	GO:0031966	C:mitochondrial membrane	0,62		1-30	311-339;468-486	311-339	468-486			
**AAEL000320**	GO:0012505	C:endomembrane system	0,94			3-26;289-317;444-462	3-26	289-317	444-462		
**AAEL000326**	GO:0012505	C:endomembrane system	0,95			3-24;300-328;458-476	3-24	300-328	458-476		
**AAEL000338**	GO:0012505	C:endomembrane system	0,95			2-21;311-339;465-483	2-21	311-339	465-483		
**AAEL000340**	GO:0012505	C:endomembrane system	0,95			2-21;289-317;441-459	2-21	289-317	441-459		
**AAEL000357**	GO:0012505	C:endomembrane system	0,94	1-18		293-321;447-464	293-321	447-464			
**AAEL002043**	GO:0012505	C:endomembrane system	0,92	1-21		289-318;443-461	289-318	443-461			
**AAEL003763**	GO:0012505	C:endomembrane system	0,95			3-26;307-335;462-480	3-26	307-335	462-480		
**AAEL004054**	GO:0012505	C:endomembrane system	0,81			11-41;354-365;367-378;505-523	11-41	354-365	367-378	505-523	
**AAEL004870**	GO:0012505	C:endomembrane system	0,93			16-42;313-340;466-484	16-42	313-340	466-484		
**AAEL004941**	GO:0012505	C:endomembrane system	0,93			2-19;295-323;456-475	2-19	295-323	456-475		
**AAEL005006**	GO:0012505	C:endomembrane system	0,93			5-35;294-322;454-473	5-35	294-322	454-473		
**AAEL005695**	GO:0012505	C:endomembrane system	0,95			2-22;291-319;445-463	2-22	291-319	445-463		
**AAEL005696**	GO:0012505	C:endomembrane system	0,95			2-21;297-325;453-471	2-21	297-325	453-471		
**AAEL005775**	GO:0012505	C:endomembrane system	0,94	1-22		295-323;449-467	295-323	449-467			
**AAEL005788**	GO:0012505	C:endomembrane system	0,95			2-20;305-333;459-477	2-20	305-333	;459-477		
**AAEL006044**	GO:0012505	C:endomembrane system	0,94	1-23		288-316;443-461	288-316	443-461			
**AAEL006058**	GO:0012505	C:endomembrane system	0,95			2-20;285-313;439-457	2-20	285-313	439-457		
**AAEL006257**	GO:0012505	C:endomembrane system	0,95			2-21;291-319;445-463	2-21	291-319	445-463		
**AAEL007473**	GO:0012505	C:endomembrane system	0,94	1-20		286-314;448-466	286-314	448-466			
**AAEL007808**	GO:0012505	C:endomembrane system	0,95			2-22;297-325;452-470	2-22	297-325	452-470		
**AAEL007815**	GO:0012505	C:endomembrane system	0,95			2-22;297-325;452-470	2-22	297-325	452-470		
**AAEL009120**	GO:0012505	C:endomembrane system	0,95			2-22;303-331;457-475	2-22	303-331	457-475		
**AAEL009123**	GO:0012505	C:endomembrane system	0,94			2-22;285-313;438-457	2-22	285-313	438-457		
**AAEL009129**	GO:0012505	C:endomembrane system	0,93			18-49;313-341;466-485	18-49	313-341	466-485		
**AAEL009130**	GO:0012505	C:endomembrane system	0,93			2-23;286-314;439-458	2-23	286-314	439-458		
**AAEL009131**	GO:0012505	C:endomembrane system	0,93			2-22;285-313;439-457	2-22	285-313	439-457		
**AAEL009132**	GO:0012505	C:endomembrane system	0,95			2-23;295-323;450-468	2-23	295-323	450-468		
**AAEL009762**	GO:0012505	C:endomembrane system	0,94			2-30;323-350;512-531	2-30	323-350	512-531		
**AAEL010154**	GO:0012505	C:endomembrane system	0,95			2-19;294-322;449-467	2-19	294-322	449-467		
**AAEL010946**	GO:0012505	C:endomembrane system	0,95			2-19;33-58;335-346;348-362;488-507	2-19	33-58	335-346	348-362	488-507
**AAEL011463**	GO:0012505	C:endomembrane system	0,81			6-28;189-200;308-336;462-481	6-28	189-200	308-336	462-481	
**AAEL011770**	GO:0012505	C:endomembrane system	0,95			2-20;286-314;436-455	2-20	286-314	436-455		
**AAEL011850**	GO:0012505	C:endomembrane system	0,82			2-14;286-314;432-450	2-14	286-314	432-450		
**AAEL012144**	GO:0012505	C:endomembrane system	0,94			2-19;295-323;444-463	2-19	295-323	444-463		
**AAEL012761**	GO:0012505	C:endomembrane system	0,95			3-26;289-317;443-462	3-26	289-317	443-462		
**AAEL012762**	GO:0012505	C:endomembrane system	0,91			222-250;377-395	222-250	377-395			
**AAEL012766**	GO:0012505	C:endomembrane system	0,95			3-22;292-320;448-466	3-22	292-320	448-466		
**AAEL012770**	GO:0012505	C:endomembrane system	0,93			2-22;298-326;453-471	2-22	298-326	453-471		
**AAEL012772**	GO:0012505	C:endomembrane system	0,95			2-22;291-319;447-465	2-22	291-319	447-465		
**AAEL013554**	GO:0012505	C:endomembrane system	0,9			32-61;337-365;494-512	32-61	337-365	494-512		
**AAEL013556**	GO:0012505	C:endomembrane system	0,94			3-25;302-330;459-477	3-25	302-330	459-477		
**AAEL014019**	GO:0012505	C:endomembrane system	0,95			2-23;298-326;454-472	2-23	298-326	454-472		
**AAEL014208**	GO:0012505	C:endomembrane system	0,95			10-32;37-60;333-361;485-503	10-32	37-60	333-361	485-503	
**AAEL014411**	GO:0012505	C:endomembrane system	0,92			3-24;308-336;461-480	3-24	308-336	461-480		
**AAEL014412**	GO:0012505	C:endomembrane system	0,93			3-24;310-338;464-482	3-24	310-338	464-482		
**AAEL014413**	GO:0012505	C:endomembrane system	0,94			2-23;313-341;467-485	2-23	313-341	467-485		
**AAEL014890**	GO:0012505	C:endomembrane system	0,93			4-28;290-318;439-457	4-28	290-318	439-457		
**AAEL017136**	GO:0012505	C:endomembrane system	0,96			2-22;290-318;443-461	2-22	290-318	443-461		
**AAEL017215**	GO:0012505	C:endomembrane system	0,94	1-20		290-318;444-462	290-318	444-462			
**AAEL017539**	GO:0012505	C:endomembrane system	0,95			2-22;283-311;437-455	2-22	283-311	437-455		
**AAEL019659**	GO:0012505	C:endomembrane system	0,95			2-22;286-314;440-457	2-22	286-314	440-457		
**AAEL022200**	GO:0012505	C:endomembrane system	0,93			2-18;288-316;442-460	2-18	288-316	442-460		
**AAEL026582**	GO:0012505	C:endomembrane system	0,93			3-24;296-324;451-469	3-24	296-324	451-469		
**AAEL026706**	GO:0012505	C:endomembrane system	0,92			2-20;290-319;444-462	2-20	290-319	444-462		
**AAEL027264**	GO:0012505	C:endomembrane system	0,94			2-18;296-324;447-465	2-18	296-324	447-465		

**AAEL029116**	GO:0012505	C:endomembrane system	0,94			2-21;294-322;448-466	2-21	294-322	448-466		
**AGAP000818**	GO:0012505	C:endomembrane system	0,95			4-31;316-344;477-495	4-31	316-344	477-495		
**AGAP000877**	GO:0012505	C:endomembrane system	0,93			16-43;356-382;509-528	16-43	356-382	509-528		
**AGAP001039**	GO:0012505	C:endomembrane system	0,94			4-25;322-350;512-531	4-25	322-350	512-531		
**AGAP001076**	GO:0012505	C:endomembrane system	0,93			13-42;352-379;507-525	13-42	352-379	507-525		
**AGAP002138**	GO:0012505	C:endomembrane system	0,94			2-19;300-328;452-470	2-19	300-328	452-470		
**AGAP002195**	GO:0012505	C:endomembrane system	0,95			3-23;297-325;452-470	3-23	297-325	452-470		
**AGAP002196**	GO:0012505	C:endomembrane system	0,95			3-24;304-332;458-476	3-24	304-332	458-476		
**AGAP002197**	GO:0012505	C:endomembrane system	0,95			3-25;298-326;453-471	3-25	298-326	453-471		
**AGAP002205**	GO:0012505	C:endomembrane system	0,95			2-21;289-317;443-461	2-21	289-317	443-461		
**AGAP002207**	GO:0012505	C:endomembrane system	0,95			2-21;289-317;443-461	2-21	289-317	443-461		
**AGAP002208**	GO:0012505	C:endomembrane system	0,93	1-26		291-319;447-465	291-319	447-465			
**AGAP002209**	GO:0012505	C:endomembrane system	0,94	1-26		291-319;447-465	291-319	447-465			
**AGAP002211**	GO:0012505	C:endomembrane system	0,93	1-26		291-319;447-465	291-319	447-465			
**AGAP002417**	GO:0012505	C:endomembrane system	0,95			2-21;287-315;438-456	2-21	287-315	438-456		
**AGAP002429**	GO:0012505	C:endomembrane system	0,94			351-379;508-527	351-379	508-527			
**AGAP002555**	GO:0012505	C:endomembrane system	0,94			2-21;299-327;451-469	2-21	299-327	451-469		
**AGAP002862**	GO:0012505	C:endomembrane system	0,94			3-24;297-325;452-470	3-24	297-325	452-470		
**AGAP002870**	GO:0012505	C:endomembrane system	0,94			3-26;289-317;446-464	3-26	289-317	446-464		
**AGAP002894**	GO:0012505	C:endomembrane system	0,93			2-22;285-313;440-458	2-22	285-313	440-458		
**AGAP003066**	GO:0012505	C:endomembrane system	0,9			3-24;310-338;463-482	3-24	310-338	463-482		
**AGAP003067**	GO:0012505	C:endomembrane system	0,94			2-22;311-339;468-486	2-22	311-339	468-486		
**AGAP003343**	GO:0012505	C:endomembrane system	0,95			2-22;285-313;445-463	2-22	285-313	445-463		
**AGAP003522**	GO:0012505	C:endomembrane system	0,94			2-27;289-317;453-471	2-27	289-317	453-471		
**AGAP005656**	GO:0012505	C:endomembrane system	0,92	1-15		289-317;447-465	289-317	447-465			
**AGAP005657**	GO:0012505	C:endomembrane system	0,91	1-21		292-321;446-464	292-321	446-464			
**AGAP005658**	GO:0012505	C:endomembrane system	0,92			2-19;289-317;443-461	2-19	289-317	443-461		
**AGAP005660**	GO:0012505	C:endomembrane system	0,91	1-21		292-321;446-464	292-321	446-464			
**AGAP006048**	GO:0012505	C:endomembrane system	0,95			5-32;321-349;478-496	5-32	321-349	478-496		
**AGAP006049**	GO:0012505	C:endomembrane system	0,93			2-22;300-328;454-472	2-22	300-328	454-472		
**AGAP007480**	GO:0012505	C:endomembrane system	0,94			2-21;284-312;445-463	2-21	284-312	445-463		
**AGAP008203**	GO:0012505	C:endomembrane system	0,94			2-21;289-318;443-461	2-21	289-318	443-461		
**AGAP008204**	GO:0012505	C:endomembrane system	0,94			2-22;290-318;443-461	2-22	290-318	443-461		
**AGAP008205**	GO:0012505	C:endomembrane system	0,96			3-25;287-314;441-459	3-25	287-314	441-459		
**AGAP008217**	GO:0012505	C:endomembrane system	0,93			2-21;286-314;440-458	2-21	286-314	440-458		
**AGAP008218**	GO:0012505	C:endomembrane system	0,93			2-22;286-314;439-458	2-22	286-314	439-458		
**AGAP008219**	GO:0012505	C:endomembrane system	0,93			2-21;286-314;440-458	2-21	286-314	440-458		
**AGAP008682**	GO:0012505	C:endomembrane system	0,95			12-33;40-63;332-360;484-502	12-33	40-63	332-360	484-502	
**AGAP009363**	GO:0012505	C:endomembrane system	0,94			2-22;317-345;475-493	2-22	317-345	475-493		
**AGAP009375**	GO:0012505	C:endomembrane system	0,95			2-21;316-344;474-492	2-21	316-344	474-492		
**AGAP009696**	GO:0012505	C:endomembrane system	0,95			15-42;351-379;504-523	15-42	351-379	504-523		
**AGAP010077**	GO:0012505	C:endomembrane system	0,91	1-21		76-87;293-322;446-464	76-87	293-322	446-464		
**AGAP010961**	GO:0012505	C:endomembrane system	0,95			5-30;308-336;470-488	5-30	308-336	470-488		
**AGAP011028**	GO:0012505	C:endomembrane system	0,95			3-23;289-317;443-461	3-23	289-317	443-461		
**AGAP011029**	GO:0012505	C:endomembrane system	0,95			3-23;289-317;443-461	3-23	289-317	443-461		
**AGAP013128**	GO:0012505	C:endomembrane system	0,93			2-22;293-321;448-466	2-22	293-321	448-466		
**AGAP013511**	GO:0012505	C:endomembrane system	0,95			2-23;266-294;427-445	2-23	266-294	427-445		
**CPIJ000293**	GO:0012505	C:endomembrane system	0,95			2-24;300-328;456-474	2-24	300-328	456-474		
**CPIJ000294**	GO:0012505	C:endomembrane system	0,93	1-23		295-323;451-469	295-323	451-469			
**CPIJ000298**	GO:0012505	C:endomembrane system	0,93	1-20		285-313;445-464	285-313	445-464			
**CPIJ000299**	GO:0012505	C:endomembrane system	0,95			2-21;288-317;448-466	2-21	288-317	448-466		
**CPIJ000655**	GO:0012505	C:endomembrane system	0,94			4-27;308-336;463-481	4-27	308-336	463-481		
**CPIJ000925**	GO:0012505	C:endomembrane system	0,94			1-21;297-325;453-471	1-21	297-325	453-471		
**CPIJ000929**	GO:0012505	C:endomembrane system	0,96			2-21;289-317;443-460	2-21	289-317	443-460		
**CPIJ000989**	GO:0012505	C:endomembrane system	0,89			3-29;35-60;328-356;480-498	3-29	35-60	328-356	480-498	
**CPIJ001038**	GO:0012505	C:endomembrane system	0,92			14-43;191-202;312-340;466-484	14-43	191-202	312-340	466-484	
**CPIJ001039**	GO:0012505	C:endomembrane system	0,77			11-35;184-195;287-315;436-455	11-35	184-195	287-315	436-455	
**CPIJ001380**	GO:0012505	C:endomembrane system	0,93			1-19;24-43;332-360;488-503	1-19	24-43	332-360	488-503	
**CPIJ001754**	GO:0012505	C:endomembrane system	0,94			2-22;298-326	2-22	298-326			
**CPIJ001755**	GO:0012505	C:endomembrane system	0,94			2-22;298-326	2-22	298-326			
**CPIJ005683**	GO:0012505	C:endomembrane system	0,95			2-21;292-320;446-464	2-21	292-320	446-464		
**CPIJ005684**	GO:0012505	C:endomembrane system	0,95			3-22;300-328;454-472	3-22	300-328	454-472		
**CPIJ005685**	GO:0012505	C:endomembrane system	0,95			2-22;299-327;453-471	2-22	299-327	453-471		
**CPIJ005954**	GO:0012505	C:endomembrane system	0,94			3-25;288-316;437-455	3-25	288-316	437-455		
**CPIJ005957**	GO:0012505	C:endomembrane system	0,95			9-36;307-335;462-480	9-36	307-335	462-480		
**CPIJ005958**	GO:0012505	C:endomembrane system	0,92			2-24;283-311;438-456	2-24	283-311	438-456		
**CPIJ005959**	GO:0012505	C:endomembrane system	0,94			3-24;297-325;452-470	3-24	297-325	452-470		
**CPIJ006950**	GO:0012505	C:endomembrane system	0,95			2-21;286-314;440-458	2-21	286-314	440-458		
**CPIJ006951**	GO:0012505	C:endomembrane system	0,91			266-294	266-294				
**CPIJ006952**	GO:0012505	C:endomembrane system	0,95			2-22;288-316;442-460	2-22	288-316	442-460		
**CPIJ007085**	GO:0012505	C:endomembrane system	0,95			2-22;294-322;448-466	2-22	294-322	448-466		
**CPIJ007086**	GO:0012505	C:endomembrane system	0,95			3-26;299-327;453-471	3-26	299-327	453-471		
**CPIJ008972**	GO:0012505	C:endomembrane system	0,93			2-23;169-180;394-412	2-23	169-180	394-412		

**CPIJ009170**	GO:0012505	C:endomembrane system	0,94			2-19;296-324;445-464	2-19	296-324	445-464		
**CPIJ009415**	GO:0012505	C:endomembrane system	0,79			14-42;353-364;366-379	14-42	353-364	366-379		
**CPIJ009473**	GO:0012505	C:endomembrane system	0,95			2-23;296-324;451-469	2-23	296-324	451-469		
**CPIJ009474**	GO:0012505	C:endomembrane system	0,95			2-22;300-328;455-473	2-22	300-328	455-473		
**CPIJ009475**	GO:0012505	C:endomembrane system	0,95			3-24;300-328;455-473	3-24	300-328	455-473		
**CPIJ009569**	GO:0012505	C:endomembrane system	0,94	1-21		281-309;435-453	281-309	435-453			
**CPIJ009570**	GO:0012505	C:endomembrane system	0,94	1-20		290-318;444-462	290-318	444-462			
**CPIJ009587**	GO:0012505	C:endomembrane system	0,69			210-238;366-384	210-238	366-384			
**CPIJ010203**	GO:0012505	C:endomembrane system	0,94			3-24;304-332;463-481	3-24	304-332	;463-481		
**CPIJ010272**	GO:0012505	C:endomembrane system	0,94	1-22		282-310;436-454	282-310	436-454			
**CPIJ010480**	GO:0012505	C:endomembrane system	0,95			3-25;303-331;460-478	3-25	303-331	460-478		
**CPIJ010810**	GO:0012505	C:endomembrane system	0,93			3-22;293-321;448-466	3-22	293-321	448-466		
**CPIJ010826**	GO:0012505	C:endomembrane system	0,86			155-166;275-303;429-448	155-166	275-303	429-448		
**CPIJ011636**	GO:0012505	C:endomembrane system	0,95			2-19;282-310;436-455	2-19	282-310	436-455		
**CPIJ011835**	GO:0012505	C:endomembrane system	0,95			2-22;298-326;452-470	2-22	298-326	452-470		
**CPIJ011837**	GO:0012505	C:endomembrane system	0,95			3-22;291-319;446-464	3-22	291-319	446-464		
**CPIJ011838**	GO:0012505	C:endomembrane system	0,95			2-22;283-311;437-456	2-22	283-311	437-456		
**CPIJ011841**	GO:0012505	C:endomembrane system	0,94			2-20;302-330;456-474	2-20	302-330	456-474		
**CPIJ011843**	GO:0012505	C:endomembrane system	0,95			2-19;277-305	2-19	277-305			
**CPIJ012470**	GO:0012505	C:endomembrane system	0,95			5-34;321-349;478-497	5-34	321-349	478-497		
**CPIJ012640**	GO:0012505	C:endomembrane system	0,95			2-22;280-308;432-450	2-22	280-308	432-450		
**CPIJ014218**	GO:0012505	C:endomembrane system	0,95			3-24;318-346;479-497	3-24	318-346	479-497		
**CPIJ014219**	GO:0012505	C:endomembrane system	0,86			21-41;150-175;470-497	21-41	150-175	470-497		
**CPIJ014220**	GO:0012505	C:endomembrane system	0,94			3-26;316-344;475-493	3-26	316-344	475-493		
**CPIJ014221**	GO:0012505	C:endomembrane system	0,96			2-22;263-291;421-440	2-22	263-291	421-440		
**CPIJ014579**	GO:0012505	C:endomembrane system	0,94	1-15		293-321;448-466	293-321	448-466			
**CPIJ014730**	GO:0012505	C:endomembrane system	0,94			3-23;293-321;447-465	3-23	293-321	447-465		
**CPIJ014944**	GO:0012505	C:endomembrane system	0,87			269-297;423-441	269-297	423-441			
**CPIJ015954**	GO:0012505	C:endomembrane system	0,87			4-28;304-332;458-477	4-28	304-332	458-477		
**CPIJ015957**	GO:0012505	C:endomembrane system	0,94			3-24;293-321;449-467	3-24	293-321	449-467		
**CPIJ015958**	GO:0012505	C:endomembrane system	0,96			3-22;296-324;451-469	3-22	296-324	451-469		
**CPIJ015959**	GO:0012505	C:endomembrane system	0,95			2-22;284-312;440-458	2-22	284-312	440-458		
**CPIJ015960**	GO:0012505	C:endomembrane system	0,94			2-22;295-323;448-466	2-22	295-323	448-466		
**CPIJ015961**	GO:0012505	C:endomembrane system	0,96			2-21;296-324;448-465	2-21	296-324	448-465		
**CPIJ015963**	GO:0012505	C:endomembrane system	0,94			8-33;288-316;439-457	8-33	288-316	439-457		
**CPIJ016284**	GO:0012505	C:endomembrane system	0,94			2-25;297-325;454-472	2-25	297-325	454-472		
**CPIJ016356**	GO:0012505	C:endomembrane system	0,94			2-21;297-325;461-479	2-21	297-325	461-479		
**CPIJ016847**	GO:0012505	C:endomembrane system	0,94			2-23;273-301;426-444	2-23	273-301	426-444		
**CPIJ016849**	GO:0012505	C:endomembrane system	0,91			2-22;173-184;287-313	2-22	173-184	287-313		
**CPIJ016851**	GO:0012505	C:endomembrane system	0,91	1-19		203-231;358-376	203-231	358-376			
**CPIJ016853**	GO:0012505	C:endomembrane system	0,92			2-22;227-255;380-399	2-22	227-255	380-399		
**CPIJ016857**	GO:0012505	C:endomembrane system	0,93	1-21		306-334;460-478	306-334	460-478			
**CPIJ017021**	GO:0012505	C:endomembrane system	0,92			6-18;231-259;384-403	6-18	231-259	384-403		
**CPIJ017199**	GO:0012505	C:endomembrane system	0,94			2-19;295-323;450-468	2-19	295-323	450-468		
**CPIJ017200**	GO:0012505	C:endomembrane system	0,85			248-276;403-421	248-276	403-421			
**CPIJ017242**	GO:0012505	C:endomembrane system	0,94			2-22;314-342;468-486	2-22	314-342	468-486		
**CPIJ017243**	GO:0012505	C:endomembrane system	0,94			3-24;308-336;461-480	3-24	308-336	461-480		
**CPIJ017244**	GO:0012505	C:endomembrane system	0,93			2-22;297-315;442-461	2-22	297-315	442-461		
**CPIJ017245**	GO:0012505	C:endomembrane system	0,9			3-25;308-336;461-480	3-25	308-336	461-480		
**CPIJ017246**	GO:0012505	C:endomembrane system	0,94			4-27;314-342;467-485	4-27	314-342	467-485		
**CPIJ017609**	GO:0012505	C:endomembrane system	0,95			6-35;295-323;456-475	6-35	295-323	456-475		
**CPIJ018494**	GO:0012505	C:endomembrane system	0,94			10-35;294-322;456-474	10-35	294-322	456-474		
**CPIJ018668**	GO:0012505	C:endomembrane system	0,93			10-38;186-197;350-378;505-523	10-38	186-197	350-378	505-523	
**CPIJ019703**	GO:0012505	C:endomembrane system	0,9			219-247;369-388;435-459	219-247	369-388	435-459		
**CPIJ020018**	GO:0012505	C:endomembrane system	0,72			49-77;397-415	49-77	397-415			
**FBgn0001992**	GO:0012505	C:endomembrane system	0,94			2-19;297-325;450-468	2-19	297-325	450-468		
**FBgn0003388**	GO:0012505	C:endomembrane system	0,92			2-34;36-47;335-363;490-508	2-34	36-47	335-363	490-508	
**FBgn0003486**	GO:0012505	C:endomembrane system	0,94			5-33;313-341;486-505	5-33	313-341	486-505		
**FBgn0004959**	GO:0012505	C:endomembrane system	0,89			10-39;357-385;507-525	10-39	357-385	507-525		
**FBgn0010019**	GO:0012505	C:endomembrane system	0,92			18-45;195-206;343-371;498-517	18-45	195-206	343-371	498-517	
**FBgn0010383**	GO:0012505	C:endomembrane system	0,94			15-44;315-343;468-486	15-44	315-343	468-486		
**FBgn0013773**	GO:0012505	C:endomembrane system	0,95			2-22;285-313;441-459	2-22	285-313	441-459		
**FBgn0015036**	GO:0012505	C:endomembrane system	0,95			2-22;291-319;445-462	2-22	291-319	445-462		
**FBgn0015037**	GO:0012505	C:endomembrane system	0,94	1-20		306-334;460-478	306-334	460-478			
**FBgn0023541**	GO:0012505	C:endomembrane system	0,91	1-15		301-329;455-473	301-329	455-473			
**FBgn0028940**	GO:0012505	C:endomembrane system	0,95			2-23;294-322;452-470	2-23	294-322	452-470		
**FBgn0030304**	GO:0012505	C:endomembrane system	0,94			10-35;519-539	10-35	519-539			
**FBgn0030339**	GO:0012505	C:endomembrane system	0,94			3-23;287-315;446-464	3-23	287-315	446-464		
**FBgn0030367**	GO:0012505	C:endomembrane system	0,95			2-20;280-308;432-450	2-20	280-308	432-450		
**FBgn0030369**	GO:0012505	C:endomembrane system	0,93			2-22;302-330;478-497	2-22	302-330	478-497		
**FBgn0030949**	GO:0012505	C:endomembrane system	0,94	1-21		273-301;432-450	273-301	432-450			
**FBgn0031126**	GO:0012505	C:endomembrane system	0,91			4-25;305-333;467-485	4-25	305-333	467-485		
**FBgn0031182**	GO:0012505	C:endomembrane system	0,92			9-42;99-110;312-340;474-492	9-42	99-110	312-340	474-492	

**FBgn0031432**	GO:0012505	C:endomembrane system	0,96			3-25;296-324;454-472	3-25	296-324	454-472		
**FBgn0031688**	GO:0012505	C:endomembrane system	0,94			3-25;292-320;448-466	3-25	292-320	448-466		
**FBgn0031689**	GO:0012505	C:endomembrane system	0,94			3-25;293-321;448-466	3-25	293-321	448-466		
**FBgn0031693**	GO:0012505	C:endomembrane system	0,95			3-31;304-332;456-474	3-31	304-332	456-474		
**FBgn0031694**	GO:0012505	C:endomembrane system	0,95			4-31;305-333;457-474	4-31	305-333	457-474		
**FBgn0031695**	GO:0012505	C:endomembrane system	0,93	1-25		303-331;455-472	303-331	455-472			
**FBgn0031925**	GO:0012505	C:endomembrane system	0,95			2-21;303-331;458-476	2-21	303-331	458-476		
**FBgn0032693**	GO:0012505	C:endomembrane system	0,91	1-19		269-297;429-447	269-297	429-447			
**FBgn0033121**	GO:0012505	C:endomembrane system	0,93			3-26;277-305;431-450	3-26	277-305	431-450		
**FBgn0033395**	GO:0012505	C:endomembrane system	0,95			3-31;312-340;466-484	3-31	312-340	466-484		
**FBgn0033397**	GO:0012505	C:endomembrane system	0,95			3-31;309-337;463-481	3-31	309-337	463-481		
**FBgn0033696**	GO:0012505	C:endomembrane system	0,95			1-23;303-331;462-480	1-23	303-331	462-480		
**FBgn0033697**	GO:0012505	C:endomembrane system	0,94			2-21;287-315;446-464	2-21	287-315	446-464		
**FBgn0033982**	GO:0012505	C:endomembrane system	0,94			2-26;307-335;463-481	2-26	307-335	463-481		
**FBgn0034756**	GO:0012505	C:endomembrane system	0,92	1-20		300-328;458-476	300-328	458-476			
**FBgn0035344**	GO:0012505	C:endomembrane system	0,91	1-27		171-182;302-330;456-473	171-182	302-330	456-473		
**FBgn0035790**	GO:0012505	C:endomembrane system	0,95			2-20;287-315;435-453	2-20	287-315	435-453		
**FBgn0036778**	GO:0012505	C:endomembrane system	0,95			2-22;303-331;457-475	2-22	303-331	457-475		
**FBgn0036910**	GO:0012505	C:endomembrane system	0,93			2-20;296-324;452-470	2-20	296-324	452-470		
**FBgn0037601**	GO:0012505	C:endomembrane system	0,94			2-21;294-322;450-469	2-21	294-322	450-469		
**FBgn0038005**	GO:0012505	C:endomembrane system	0,95			2-21;279-307;435-453	2-21	279-307	435-453		
**FBgn0038006**	GO:0012505	C:endomembrane system	0,95			2-20;284-312;440-458	2-20	284-312	440-458		
**FBgn0038007**	GO:0012505	C:endomembrane system	0,95			2-21;284-312;440-458	2-21	284-312	440-458		
**FBgn0038076**	GO:0012505	C:endomembrane system	0,95			2-21;286-314;442-460	2-21	286-314	442-460		
**FBgn0038095**	GO:0012505	C:endomembrane system	0,93			2-20;302-330;456-474	2-20	302-330	456-474		
**FBgn0038194**	GO:0012505	C:endomembrane system	0,95			2-21;301-329;455-473	2-21	301-329	455-473		
**FBgn0038236**	GO:0012505	C:endomembrane system	0,95			2-21;284-312;440-458	2-21	284-312	440-458		
**FBgn0039006**	GO:0012505	C:endomembrane system	0,92			2-22;305-333;459-477	2-22	305-333	459-477		
**FBgn0086917**	GO:0012505	C:endomembrane system	0,94			6-38;309-336;501-520	6-38	309-336	501-520		

Culled “labile” CYPs (BUSCA output)

CYP ID	GOids	GOterms	Score	Signal Peptide	Mitochondrion Transit Peptide	Transmembrane Alpha Helix	1st TMH	2nd TMH	3rd TMH	4th TMH
**AGAP008022**	GO:0031090	C:organelle membrane	0,89			311-339;471-489	311-339	471-489		
**FBgn0034387**	GO:0031090	C:organelle membrane	0,8			318-346;480-499	318-346	480-499		
**FBgn0036806**	GO:0031090	C:organelle membrane	0,88			310-338;472-490	310-338	472-490		
**AAEL001960**	GO:0031966	C:mitochondrial membrane	0,69		1-29	315-343;474-492	315-343	474-492		
**AAEL006827**	GO:0031966	C:mitochondrial membrane	0,63		1-25	301-329;458-476	301-329	458-476		
**AGAP008019**	GO:0031966	C:mitochondrial membrane	0,7		1-30	312-340;471-489	312-340	471-489		
**AGAP008020**	GO:0031966	C:mitochondrial membrane	0,59		1-28	311-339;470-488	311-339	470-488		
**CPIJ010227**	GO:0031966	C:mitochondrial membrane	0,72		1-34	315-343;473-491	315-343	473-491		
**FBgn0033524**	GO:0031966	C:mitochondrial membrane	0,6		1-14	375-403;537-555	375-403	537-555		
**FBgn0038681**	GO:0031966	C:mitochondrial membrane	0,72		1-32	319-347;483-501	319-347	483-501		
**AAEL001292**	GO:0012505	C:endomembrane system	0,94			2-24;317-345;476-494	2-24	317-345	476-494	
**AAEL001320**	GO:0012505	C:endomembrane system	0,95			3-23;315-343;474-492	3-23	315-343	474-492	
**AAEL002085**	GO:0012505	C:endomembrane system	0,95			2-20;298-326;453-471	2-20	298-326	453-471	
**AAEL003380**	GO:0012505	C:endomembrane system	0,95			11-39;315-343;471-489	11-39	315-343	471-489	
**AAEL003748**	GO:0012505	C:endomembrane system	0,94			2-23;313-341;472-490	2-23	313-341	472-490	
**AAEL006805**	GO:0012505	C:endomembrane system	0,94			3-25;321-349;480-498	3-25	321-349	480-498	
**AAEL006989**	GO:0012505	C:endomembrane system	0,95			2-22;284-312;444-462	2-22	284-312	444-462	
**AAEL007795**	GO:0012505	C:endomembrane system	0,93			17-49;197-208;319-347;474-492	17-49	197-208	319-347	474-492
**AAEL007812**	GO:0012505	C:endomembrane system	0,95			2-21;293-321;449-467	2-21	293-321	449-467	
**AAEL007830**	GO:0012505	C:endomembrane system	0,95			3-28;306-334;461-479	3-28	306-334	461-479	
**AAEL008018**	GO:0012505	C:endomembrane system	0,95			23-54;339-367;493-511	23-54	339-367	493-511	
**AAEL008889**	GO:0012505	C:endomembrane system	0,95			2-21;298-326;453-471	2-21	298-326	453-471	
**AAEL009018**	GO:0012505	C:endomembrane system	0,95			2-20;296-324;447-465	2-20	296-324	447-465	
**AAEL009125**	GO:0012505	C:endomembrane system	0,95			2-23;285-313;440-458	2-23	285-313	440-458	
**AAEL009126**	GO:0012505	C:endomembrane system	0,93			2-20;291-319;445-463	2-20	291-319	445-463	
**AAEL009133**	GO:0012505	C:endomembrane system	0,93			2-20;290-318;444-462	2-20	290-318	444-462	
**AAEL009591**	GO:0012505	C:endomembrane system	0,89			29-57;114-125;354-382;512-531	29-57	114-125	354-382	512-531
**AAEL009656**	GO:0012505	C:endomembrane system	0,95			2-21;300-328;450-469	2-21	300-328	450-469	
**AAEL011761**	GO:0012505	C:endomembrane system	0,9			3-23;299-327;454-472	3-23	299-327	454-472	
**AAEL012765**	GO:0012505	C:endomembrane system	0,95			3-23;291-319;446-464	3-23	291-319	446-464	
**AAEL014603**	GO:0012505	C:endomembrane system	0,93			3-23;324-352;483-501	3-23	324-352	483-501	
**AAEL014614**	GO:0012505	C:endomembrane system	0,95			3-24;327-355;486-504	3-24	327-355	486-504	
**AAEL014615**	GO:0012505	C:endomembrane system	0,94			3-24;318-346;477-495	3-24	318-346	477-495	
**AAEL014617**	GO:0012505	C:endomembrane system	0,95			3-23;322-350;481-499	3-23	322-350	481-499	
**AAEL014684**	GO:0012505	C:endomembrane system	0,95			2-24;299-327;451-469	2-24	299-327	451-469	
**AAEL014893**	GO:0012505	C:endomembrane system	0,93			3-23;297-325;452-470	3-23	297-325	452-470	
**AAEL019603**	GO:0012505	C:endomembrane system	0,95			3-23;324-352;482-500	3-23	324-352	482-500	
**AAEL022568**	GO:0012505	C:endomembrane system	0,94			2-23;294-322;449-467	2-23	294-322	449-467	
**AGAP000088**	GO:0012505	C:endomembrane system	0,95			2-19;302-330;457-475	2-19	302-330	457-475	
**AGAP000193**	GO:0012505	C:endomembrane system	0,87			23-53;206-217;339-367;496-514	23-53	206-217	339-367	496-514
**AGAP000194**	GO:0012505	C:endomembrane system	0,95			10-41;348-376;501-520	10-41	348-376	501-520	
**AGAP002206**	GO:0012505	C:endomembrane system	0,95			3-27;289-317;443-461	3-27	289-317	443-461	
**AGAP002210**	GO:0012505	C:endomembrane system	0,95			2-20;291-319;446-464	2-20	291-319	446-464	
**AGAP002419**	GO:0012505	C:endomembrane system	0,94			2-23;300-328;455-473	2-23	300-328	455-473	
**AGAP002865**	GO:0012505	C:endomembrane system	0,94			3-24;301-329;456-474	3-24	301-329	456-474	
**AGAP002868**	GO:0012505	C:endomembrane system	0,94			2-24;301-329;455-474	2-24	301-329	455-474	
**AGAP002869**	GO:0012505	C:endomembrane system	0,93			2-25;299-327;454-472	2-25	299-327	454-472	
**AGAP003608**	GO:0012505	C:endomembrane system	0,92			8-35;290-318;442-461	8-35	290-318	442-461	
**AGAP006047**	GO:0012505	C:endomembrane system	0,94			2-23;298-326;454-472	2-23	298-326	454-472	
**AGAP008206**	GO:0012505	C:endomembrane system	0,94			2-21;292-320;446-464	2-21	292-320	446-464	
**AGAP008207**	GO:0012505	C:endomembrane system	0,94			2-29;302-330;457-475	2-29	302-330	457-475	
**AGAP008208**	GO:0012505	C:endomembrane system	0,95			3-24;294-322;449-467	3-24	294-322	449-467	
**AGAP008209**	GO:0012505	C:endomembrane system	0,93			3-26;289-318;445-463	3-26	289-318	445-463	
**AGAP008214**	GO:0012505	C:endomembrane system	0,95			3-24;290-318;445-463	3-24	290-318	445-463	
**AGAP008552**	GO:0012505	C:endomembrane system	0,92			16-48;198-209;327-355;482-500	16-48	198-209	327-355	482-500
**AGAP009240**	GO:0012505	C:endomembrane system	0,95			4-30;312-340;466-484	4-30	312-340	466-484	
**AGAP010414**	GO:0012505	C:endomembrane system	0,94			11-39;324-352;478-496	11-39	324-352	478-496	
**AGAP012291**	GO:0012505	C:endomembrane system	0,94			3-24;334-362;494-512	3-24	334-362	494-512	
**AGAP012292**	GO:0012505	C:endomembrane system	0,94			3-24;325-353;484-502	3-24	325-353	484-502	
**AGAP012294**	GO:0012505	C:endomembrane system	0,94			3-23;322-350;482-500	3-23	322-350	482-500	
**AGAP012296**	GO:0012505	C:endomembrane system	0,94			3-24;328-356;487-505	3-24	328-356	487-505	
**AGAP013224**	GO:0012505	C:endomembrane system	0,95			4-27;308-336;454-473	4-27	308-336	454-473	
**AGAP013241**	GO:0012505	C:endomembrane system	0,95			3-23;302-330;457-475	3-23	302-330	457-475	

**CPIJ001757**	GO:0012505	C:endomembrane system	0,94	1-15		298-326;453-471	298-326	453-471		
**CPIJ002535**	GO:0012505	C:endomembrane system	0,94			2-24;285-313;447-465	2-24	285-313	447-465	
**CPIJ002538**	GO:0012505	C:endomembrane system	0,95			3-24;287-315;446-464	3-24	287-315	446-464	
**CPIJ003082**	GO:0012505	C:endomembrane system	0,95			3-23;320-348;479-497	3-23	320-348	479-497	
**CPIJ003361**	GO:0012505	C:endomembrane system	0,94			2-22;284-312;438-456	2-22	284-312	438-456	
**CPIJ003377**	GO:0012505	C:endomembrane system	0,95			3-23;282-310;436-454	3-23	282-310	436-454	
**CPIJ004411**	GO:0012505	C:endomembrane system	0,93			2-22;283-311;436-455	2-22	283-311	436-455	
**CPIJ005952**	GO:0012505	C:endomembrane system	0,95			2-24;290-318;445-463	2-24	290-318	445-463	
**CPIJ005953**	GO:0012505	C:endomembrane system	0,94			3-24;297-325;452-470	3-24	297-325	452-470	
**CPIJ005955**	GO:0012505	C:endomembrane system	0,95			2-24;305-333;460-478	2-24	305-333	460-478	
**CPIJ005956**	GO:0012505	C:endomembrane system	0,93			4-28;297-326;451-469	4-28	297-326	451-469	
**CPIJ006721**	GO:0012505	C:endomembrane system	0,95			2-21;299-327;457-475	2-21	299-327	457-475	
**CPIJ007089**	GO:0012505	C:endomembrane system	0,71			287-315;443-461	287-315	443-461		
**CPIJ007091**	GO:0012505	C:endomembrane system	0,95			2-22;292-320;446-464	2-22	292-320	446-464	
**CPIJ007095**	GO:0012505	C:endomembrane system	0,95			2-20;291-319;445-463	2-20	291-319	445-463	
**CPIJ009468**	GO:0012505	C:endomembrane system	0,95			2-22;294-322;447-466	2-22	294-322	447-466	
**CPIJ010175**	GO:0012505	C:endomembrane system	0,94			2-23;322-350;480-498	2-23	322-350	480-498	
**CPIJ010536**	GO:0012505	C:endomembrane system	0,95			3-25;326-354;485-503	3-25	326-354	485-503	
**CPIJ010538**	GO:0012505	C:endomembrane system	0,94			2-24;323-351;482-500	2-24	323-351	482-500	
**CPIJ010545**	GO:0012505	C:endomembrane system	0,94			3-24;317-345;476-494	3-24	317-345	476-494	
**CPIJ010547**	GO:0012505	C:endomembrane system	0,95			3-23;324-352;483-501	3-23	324-352	483-501	
**CPIJ010858**	GO:0012505	C:endomembrane system	0,95			2-23;297-325;450-468	2-23	297-325	450-468	
**CPIJ011127**	GO:0012505	C:endomembrane system	0,94	1-18		295-323;451-469	295-323	451-469		
**CPIJ011129**	GO:0012505	C:endomembrane system	0,93			2-20;288-316;442-460	2-20	288-316	442-460	
**CPIJ011836**	GO:0012505	C:endomembrane system	0,92	1-21		146-157;258-287;412-430	146-157	258-287	412-430	
**CPIJ014940**	GO:0012505	C:endomembrane system	0,9	1-21		72-83;278-307;429-447	72-83	278-307	429-447	
**CPIJ014941**	GO:0012505	C:endomembrane system	0,93			2-17;285-314;436-454	2-17	285-314	436-454	
**CPIJ014942**	GO:0012505	C:endomembrane system	0,93	1-21		285-313;434-454	285-313	434-454		
**CPIJ015428**	GO:0012505	C:endomembrane system	0,94			2-22;286-314;440-458	2-22	286-314	440-458	
**CPIJ016852**	GO:0012505	C:endomembrane system	0,94			2-21;291-319;445-463	2-21	291-319	445-463	
**CPIJ016855**	GO:0012505	C:endomembrane system	0,94			2-21;291-319;445-463	2-21	291-319	445-463	
**CPIJ017462**	GO:0012505	C:endomembrane system	0,92			2-20;278-306;432-450	2-20	278-306	432-450	
**CPIJ019587**	GO:0012505	C:endomembrane system	0,93			2-21;282-310;435-453	2-21	282-310	435-453	
**CPIJ019673**	GO:0012505	C:endomembrane system	0,94			2-22;284-312;443-462	2-22	284-312	443-462	
**FBgn0000473**	GO:0012505	C:endomembrane system	0,93	1-16		296-324;452-470	296-324	452-470		
**FBgn0011576**	GO:0012505	C:endomembrane system	0,95			2-22;298-326;451-469	2-22	298-326	451-469	
**FBgn0013771**	GO:0012505	C:endomembrane system	0,94			3-23;295-323;451-469	3-23	295-323	451-469	
**FBgn0015034**	GO:0012505	C:endomembrane system	0,96			2-22;293-322;446-463	2-22	293-322	446-463	
**FBgn0015039**	GO:0012505	C:endomembrane system	0,95			2-22;291-319;451-469	2-22	291-319	451-469	
**FBgn0015040**	GO:0012505	C:endomembrane system	0,95			2-24;306-334;466-484	2-24	306-334	466-484	
**FBgn0025454**	GO:0012505	C:endomembrane system	0,93			3-23;299-327;460-478	3-23	299-327	460-478	
**FBgn0030615**	GO:0012505	C:endomembrane system	0,95			2-24;293-322;438-455	2-24	293-322	438-455	
**FBgn0033065**	GO:0012505	C:endomembrane system	0,93	1-18		291-319;451-469	291-319	451-469		
**FBgn0033292**	GO:0012505	C:endomembrane system	0,95			2-23;292-320;447-465	2-23	292-320	447-465	
**FBgn0033302**	GO:0012505	C:endomembrane system	0,95			2-23;300-328;456-474	2-23	300-328	456-474	
**FBgn0033304**	GO:0012505	C:endomembrane system	0,95			2-22;282-310;437-455	2-22	282-310	437-455	
**FBgn0033775**	GO:0012505	C:endomembrane system	0,94			3-24;306-334;463-482	3-24	306-334	463-482	
**FBgn0033978**	GO:0012505	C:endomembrane system	0,94			2-21;292-320;447-465	2-21	292-320	447-465	
**FBgn0033980**	GO:0012505	C:endomembrane system	0,94			2-22;292-320;447-465	2-22	292-320	447-465	
**FBgn0034053**	GO:0012505	C:endomembrane system	0,94			8-36;298-327;452-470	8-36	298-327	452-470	
**FBgn0038037**	GO:0012505	C:endomembrane system	0,93	1-22		303-331;461-479	303-331	461-479		
**FBgn0039519**	GO:0012505	C:endomembrane system	0,92			2-25;297-325;453-471	2-25	297-325	453-471	

Culled “stable” CYPs (BUSCA output)

CYP IDs	GOids	GOterms	Score	Signal Peptide	Mitochondrion Transit Peptide	Transmembrane Alpha Helix	1st TMH	2nd TMH	3rd TMH	4th TMH
**CPIJ005899**	GO:0005886	C:plasma membrane	0,73			114-139;422-450	114-139	422-450		
**AAEL005700**	GO:0031090	C:organelle membrane	0,7			8-36;306-334;459-477	8-36	306-334	459-477	
**AAEL014594**	GO:0031090	C:organelle membrane	0,91			375-403;532-550	375-403	532-550		
**AGAP000284**	GO:0031090	C:organelle membrane	0,94			288-316;433-452	288-316	433-452		
**AGAP005992**	GO:0031090	C:organelle membrane	0,87			309-337;465-483	309-337	465-483		
**FBgn0000449**	GO:0031090	C:organelle membrane	0,81			283-311;435-454	283-311	435-454		
**FBgn0003312**	GO:0031090	C:organelle membrane	0,89			10-39;328-356;468-486	10-39	328-356	468-486	
**FBgn0037817**	GO:0031090	C:organelle membrane	0,88			4-21;310-338;470-488	4-21	310-338	470-488	
**AGAP008018**	GO:0031966	C:mitochondrial membrane	0,76		1-22	310-337;469-487	310-337	469-487		
**FBgn0053503**	GO:0031966	C:mitochondrial membrane	0,62		1-30	311-339;468-486	311-339	468-486		
**AAEL000320**	GO:0012505	C:endomembrane system	0,94			3-26;289-317;444-462	3-26	289-317	444-462	
**AAEL000326**	GO:0012505	C:endomembrane system	0,95			3-24;300-328;458-476	3-24	300-328	458-476	
**AAEL000338**	GO:0012505	C:endomembrane system	0,95			2-21;311-339;465-483	2-21	311-339	465-483	
**AAEL000340**	GO:0012505	C:endomembrane system	0,95			2-21;289-317;441-459	2-21	289-317	441-459	
**AAEL000357**	GO:0012505	C:endomembrane system	0,94	1-18		293-321;447-464	293-321	447-464		
**AAEL005006**	GO:0012505	C:endomembrane system	0,93			5-35;294-322;454-473	5-35	294-322	454-473	
**AAEL005695**	GO:0012505	C:endomembrane system	0,95			2-22;291-319;445-463	2-22	291-319	445-463	
**AAEL005696**	GO:0012505	C:endomembrane system	0,95			2-21;297-325;453-471	2-21	297-325	453-471	
**AAEL005775**	GO:0012505	C:endomembrane system	0,94	1-22		295-323;449-467	295-323	449-467		
**AAEL005788**	GO:0012505	C:endomembrane system	0,95			2-20;305-333;459-477	2-20	305-333	459-477	
**AAEL006044**	GO:0012505	C:endomembrane system	0,94	1-23		288-316;443-461	288-316	443-461		
**AAEL006058**	GO:0012505	C:endomembrane system	0,95			2-20;285-313;439-457	2-20	285-313	439-457	
**AAEL006257**	GO:0012505	C:endomembrane system	0,95			2-21;291-319;445-463	2-21	291-319	445-463	
**AAEL009129**	GO:0012505	C:endomembrane system	0,93			18-49;313-341;466-485	18-49	313-341	466-485	
**AAEL011770**	GO:0012505	C:endomembrane system	0,95			2-20;286-314;436-455	2-20	286-314	436-455	
**AAEL011850**	GO:0012505	C:endomembrane system	0,82			2-14;286-314;432-450	2-14	286-314	432-450	
**AAEL012761**	GO:0012505	C:endomembrane system	0,95			3-26;289-317;443-462	3-26	289-317	443-462	
**AAEL012762**	GO:0012505	C:endomembrane system	0,91			222-250;377-395	222-250	377-395		
**AAEL012766**	GO:0012505	C:endomembrane system	0,95			3-22;292-320;448-466	3-22	292-320	448-466	
**AAEL012770**	GO:0012505	C:endomembrane system	0,93			2-22;298-326;453-471	2-22	298-326	453-471	
**AAEL013554**	GO:0012505	C:endomembrane system	0,9			32-61;337-365;494-512	32-61	337-365	494-512	
**AAEL014019**	GO:0012505	C:endomembrane system	0,95			2-23;298-326;454-472	2-23	298-326	454-472	
**AAEL014890**	GO:0012505	C:endomembrane system	0,93			4-28;290-318;439-457	4-28	290-318	439-457	
**AAEL017136**	GO:0012505	C:endomembrane system	0,96			2-22;290-318;443-461	2-22	290-318	443-461	
**AAEL017215**	GO:0012505	C:endomembrane system	0,94	1-20		290-318;444-462	290-318	444-462		
**AAEL017539**	GO:0012505	C:endomembrane system	0,95			2-22;283-311;437-455	2-22	283-311	437-455	
**AAEL022200**	GO:0012505	C:endomembrane system	0,93			2-18;288-316;442-460	2-18	288-316	442-460	
**AAEL026706**	GO:0012505	C:endomembrane system	0,92			2-20;290-319;444-462	2-20	290-319	444-462	
**AAEL027264**	GO:0012505	C:endomembrane system	0,94			2-18;296-324;447-465	2-18	296-324	447-465	
**AGAP000818**	GO:0012505	C:endomembrane system	0,95			4-31;316-344;477-495	4-31	316-344	477-495	
**AGAP000877**	GO:0012505	C:endomembrane system	0,93			16-43;356-382;509-528	16-43	356-382	509-528	
**AGAP001039**	GO:0012505	C:endomembrane system	0,94			4-25;322-350;512-531	4-25	322-350	512-531	
**AGAP002138**	GO:0012505	C:endomembrane system	0,94			2-19;300-328;452-470	2-19	300-328	452-470	
**AGAP002195**	GO:0012505	C:endomembrane system	0,95			3-23;297-325;452-470	3-23	297-325	452-470	
**AGAP002196**	GO:0012505	C:endomembrane system	0,95			3-24;304-332;458-476	3-24	304-332	458-476	
**AGAP002209**	GO:0012505	C:endomembrane system	0,94	1-26		291-319;447-465	291-319	447-465		
**AGAP002417**	GO:0012505	C:endomembrane system	0,95			2-21;287-315;438-456	2-21	287-315	438-456	
**AGAP002429**	GO:0012505	C:endomembrane system	0,94			351-379;508-527	351-379	508-527		
**AGAP002555**	GO:0012505	C:endomembrane system	0,94			2-21;299-327;451-469	2-21	299-327	451-469	
**AGAP002862**	GO:0012505	C:endomembrane system	0,94			3-24;297-325;452-470	3-24	297-325	452-470	
**AGAP002870**	GO:0012505	C:endomembrane system	0,94			3-26;289-317;446-464	3-26	289-317	446-464	
**AGAP002894**	GO:0012505	C:endomembrane system	0,93			2-22;285-313;440-458	2-22	285-313	440-458	
**AGAP003066**	GO:0012505	C:endomembrane system	0,9			3-24;310-338;463-482	3-24	310-338	463-482	
**AGAP003343**	GO:0012505	C:endomembrane system	0,95			2-22;285-313;445-463	2-22	285-313	445-463	
**AGAP003522**	GO:0012505	C:endomembrane system	0,94			2-27;289-317;453-471	2-27	289-317	453-471	
**AGAP005658**	GO:0012505	C:endomembrane system	0,92			2-19;289-317;443-461	2-19	289-317	443-461	
**AGAP005660**	GO:0012505	C:endomembrane system	0,91	1-21		292-321;446-464	292-321	446-464		
**AGAP006048**	GO:0012505	C:endomembrane system	0,95			5-32;321-349;478-496	5-32	321-349	478-496	
**AGAP006049**	GO:0012505	C:endomembrane system	0,93			2-22;300-328;454-472	2-22	300-328	454-472	
**AGAP007480**	GO:0012505	C:endomembrane system	0,94			2-21;284-312;445-463	2-21	284-312	445-463	
**AGAP008205**	GO:0012505	C:endomembrane system	0,96			3-25;287-314;441-459	3-25	287-314	441-459	
**AGAP008682**	GO:0012505	C:endomembrane system	0,95			12-33;40-63;332-360;484-502	12-33	40-63	332-360	484-502

**AGAP009375**	GO:0012505	C:endomembrane system	0,95			2-21;316-344;474-492	2-21	316-344	474-492	
**AGAP009696**	GO:0012505	C:endomembrane system	0,95			15-42;351-379;504-523	15-42	351-379	504-523	
**AGAP010961**	GO:0012505	C:endomembrane system	0,95			5-30;308-336;470-488	5-30	308-336	470-488	
**AGAP011029**	GO:0012505	C:endomembrane system	0,95			3-23;289-317;443-461	3-23	289-317	443-461	
**AGAP013511**	GO:0012505	C:endomembrane system	0,95			2-23;266-294;427-445	2-23	266-294	427-445	
**CPIJ000293**	GO:0012505	C:endomembrane system	0,95			2-24;300-328;456-474	2-24	300-328	456-474	
**CPIJ000294**	GO:0012505	C:endomembrane system	0,93	1-23		295-323;451-469	295-323	451-469		
**CPIJ000298**	GO:0012505	C:endomembrane system	0,93	1-20		285-313;445-464	285-313	445-464		
**CPIJ000299**	GO:0012505	C:endomembrane system	0,95			2-21;288-317;448-466	2-21	288-317	448-466	
**CPIJ000655**	GO:0012505	C:endomembrane system	0,94			4-27;308-336;463-481	4-27	308-336	463-481	
**CPIJ000925**	GO:0012505	C:endomembrane system	0,94			1-21;297-325;453-471	1-21	297-325	453-471	
**CPIJ005683**	GO:0012505	C:endomembrane system	0,95			2-21;292-320;446-464	2-21	292-320	446-464	
**CPIJ005684**	GO:0012505	C:endomembrane system	0,95			3-22;300-328;454-472	3-22	300-328	454-472	
**CPIJ005954**	GO:0012505	C:endomembrane system	0,94			3-25;288-316;437-455	3-25	288-316	437-455	
**CPIJ005957**	GO:0012505	C:endomembrane system	0,95			9-36;307-335;462-480	9-36	307-335	462-480	
**CPIJ006952**	GO:0012505	C:endomembrane system	0,95			2-22;288-316;442-460	2-22	288-316	442-460	
**CPIJ007085**	GO:0012505	C:endomembrane system	0,95			2-22;294-322;448-466	2-22	294-322	448-466	
**CPIJ007086**	GO:0012505	C:endomembrane system	0,95			3-26;299-327;453-471	3-26	299-327	453-471	
**CPIJ008972**	GO:0012505	C:endomembrane system	0,93			2-23;169-180;394-412	2-23	169-180	394-412	
**CPIJ009170**	GO:0012505	C:endomembrane system	0,94			2-19;296-324;445-464	2-19	296-324	445-464	
**CPIJ009473**	GO:0012505	C:endomembrane system	0,95			2-23;296-324;451-469	2-23	296-324	451-469	
**CPIJ009569**	GO:0012505	C:endomembrane system	0,94	1-21		281-309;435-453	281-309	435-453		
**CPIJ009570**	GO:0012505	C:endomembrane system	0,94	1-20		290-318;444-462	290-318	444-462		
**CPIJ010203**	GO:0012505	C:endomembrane system	0,94			3-24;304-332;463-481	3-24	304-332	463-481	
**CPIJ010272**	GO:0012505	C:endomembrane system	0,94	1-22		282-310;436-454	282-310	436-454		
**CPIJ010810**	GO:0012505	C:endomembrane system	0,93			3-22;293-321;448-466	3-22	293-321	448-466	
**CPIJ011835**	GO:0012505	C:endomembrane system	0,95			2-22;298-326;452-470	2-22	298-326	452-470	
**CPIJ011837**	GO:0012505	C:endomembrane system	0,95			3-22;291-319;446-464	3-22	291-319	446-464	
**CPIJ011838**	GO:0012505	C:endomembrane system	0,95			2-22;283-311;437-456	2-22	283-311	437-456	
**CPIJ011843**	GO:0012505	C:endomembrane system	0,95			2-19;277-305	2-19	277-305		
**CPIJ012470**	GO:0012505	C:endomembrane system	0,95			5-34;321-349;478-497	5-34	321-349	478-497	
**CPIJ012640**	GO:0012505	C:endomembrane system	0,95			2-22;280-308;432-450	2-22	280-308	432-450	
**CPIJ014218**	GO:0012505	C:endomembrane system	0,95			3-24;318-346;479-497	3-24	318-346	479-497	
**CPIJ014219**	GO:0012505	C:endomembrane system	0,86			21-41;150-175;470-497	21-41	150-175	470-497	
**CPIJ014220**	GO:0012505	C:endomembrane system	0,94			3-26;316-344;475-493	3-26	316-344	475-493	
**CPIJ014221**	GO:0012505	C:endomembrane system	0,96			2-22;263-291;421-440	2-22	263-291	421-440	
**CPIJ014579**	GO:0012505	C:endomembrane system	0,94	1-15		293-321;448-466	293-321	448-466		
**CPIJ014730**	GO:0012505	C:endomembrane system	0,94			3-23;293-321;447-465	3-23	293-321	447-465	
**CPIJ015954**	GO:0012505	C:endomembrane system	0,87			4-28;304-332;458-477	4-28	304-332	458-477	
**CPIJ015958**	GO:0012505	C:endomembrane system	0,96			3-22;296-324;451-469	3-22	296-324	451-469	
**CPIJ015959**	GO:0012505	C:endomembrane system	0,95			2-22;284-312;440-458	2-22	284-312	440-458	
**CPIJ015960**	GO:0012505	C:endomembrane system	0,94			2-22;295-323;448-466	2-22	295-323	448-466	
**CPIJ015961**	GO:0012505	C:endomembrane system	0,96			2-21;296-324;448-465	2-21	296-324	448-465	
**CPIJ015963**	GO:0012505	C:endomembrane system	0,94			8-33;288-316;439-457	8-33	288-316	439-457	
**CPIJ016356**	GO:0012505	C:endomembrane system	0,94			2-21;297-325;461-479	2-21	297-325	461-479	
**CPIJ016847**	GO:0012505	C:endomembrane system	0,94			2-23;273-301;426-444	2-23	273-301	426-444	
**CPIJ016849**	GO:0012505	C:endomembrane system	0,91			2-22;173-184;287-313	2-22	173-184	287-313	
**CPIJ016853**	GO:0012505	C:endomembrane system	0,92			2-22;227-255;380-399	2-22	227-255	380-399	
**CPIJ016857**	GO:0012505	C:endomembrane system	0,93	1-21		306-334;460-478	306-334	460-478		
**CPIJ017199**	GO:0012505	C:endomembrane system	0,94			2-19;295-323;450-468	2-19	295-323	450-468	
**CPIJ017242**	GO:0012505	C:endomembrane system	0,94			2-22;314-342;468-486	2-22	314-342	468-486	
**CPIJ017245**	GO:0012505	C:endomembrane system	0,9			3-25;308-336;461-480	3-25	308-336	461-480	
**CPIJ017246**	GO:0012505	C:endomembrane system	0,94			4-27;314-342;467-485	4-27	314-342	467-485	
**CPIJ017609**	GO:0012505	C:endomembrane system	0,95			6-35;295-323;456-475	6-35	295-323	456-475	
**CPIJ019703**	GO:0012505	C:endomembrane system	0,9			219-247;369-388;435-459	219-247	369-388	435-459	
**CPIJ020018**	GO:0012505	C:endomembrane system	0,72			49-77;397-415	49-77	397-415		
**FBgn0001992**	GO:0012505	C:endomembrane system	0,94			2-19;297-325;450-468	2-19	297-325	450-468	
**FBgn0003388**	GO:0012505	C:endomembrane system	0,92			2-34;36-47;335-363;490-508	2-34	36-47	335-363	490-508
**FBgn0003486**	GO:0012505	C:endomembrane system	0,94			5-33;313-341;486-505	5-33	313-341	486-505	
**FBgn0004959**	GO:0012505	C:endomembrane system	0,89			10-39;357-385;507-525	10-39	357-385	507-525	
**FBgn0010019**	GO:0012505	C:endomembrane system	0,92			18-45;195-206;343-371;498-51	18-45	195-206	343-371	498-517
**FBgn0010383**	GO:0012505	C:endomembrane system	0,94			15-44;315-343;468-486	15-44	315-343	468-486	
**FBgn0013773**	GO:0012505	C:endomembrane system	0,95			2-22;285-313;441-459	2-22	285-313	441-459	
**FBgn0015036**	GO:0012505	C:endomembrane system	0,95			2-22;291-319;445-462	2-22	291-319	445-462	
**FBgn0023541**	GO:0012505	C:endomembrane system	0,91	1-15		301-329;455-473	301-329	455-473		

**FBgn0028940**	GO:0012505	C:endomembrane system	0,95			2-23;294-322;452-470	2-23	294-322	452-470	
**FBgn0030304**	GO:0012505	C:endomembrane system	0,94			10-35;519-539	10-35	519-539		
**FBgn0030339**	GO:0012505	C:endomembrane system	0,94			3-23;287-315;446-464	3-23	287-315	446-464	
**FBgn0030367**	GO:0012505	C:endomembrane system	0,95			2-20;280-308;432-450	2-20	280-308	432-450	
**FBgn0030369**	GO:0012505	C:endomembrane system	0,93			2-22;302-330;478-497	2-22	302-330	478-497	
**FBgn0030949**	GO:0012505	C:endomembrane system	0,94	1-21		273-301;432-450	273-301	432-450		
**FBgn0031126**	GO:0012505	C:endomembrane system	0,91			4-25;305-333;467-485	4-25	305-333	467-485	
**FBgn0031182**	GO:0012505	C:endomembrane system	0,92			9-42;99-110;312-340;474-492	9-42	99-110	312-340	474-492
**FBgn0031432**	GO:0012505	C:endomembrane system	0,96			3-25;296-324;454-472	3-25	296-324	454-472	
**FBgn0031689**	GO:0012505	C:endomembrane system	0,94			3-25;293-321;448-466	3-25	293-321	448-466	
**FBgn0031694**	GO:0012505	C:endomembrane system	0,95			4-31;305-333;457-474	4-31	305-333	457-474	
**FBgn0031925**	GO:0012505	C:endomembrane system	0,95			2-21;303-331;458-476	2-21	303-331	458-476	
**FBgn0032693**	GO:0012505	C:endomembrane system	0,91	1-19		269-297;429-447	269-297	429-447		
**FBgn0033121**	GO:0012505	C:endomembrane system	0,93			3-26;277-305;431-450	3-26	277-305	431-450	
**FBgn0033395**	GO:0012505	C:endomembrane system	0,95			3-31;312-340;466-484	3-31	312-340	466-484	
**FBgn0033696**	GO:0012505	C:endomembrane system	0,95			1-23;303-331;462-480	1-23	303-331	462-480	
**FBgn0033697**	GO:0012505	C:endomembrane system	0,94			2-21;287-315;446-464	2-21	287-315	446-464	
**FBgn0033982**	GO:0012505	C:endomembrane system	0,94			2-26;307-335;463-481	2-26	307-335	463-481	
**FBgn0034756**	GO:0012505	C:endomembrane system	0,92	1-20		300-328;458-476	300-328	458-476		
**FBgn0035344**	GO:0012505	C:endomembrane system	0,91	1-27		171-182;302-330;456-473	171-182	302-330	456-473	
**FBgn0035790**	GO:0012505	C:endomembrane system	0,95			2-20;287-315;435-453	2-20	287-315	435-453	
**FBgn0036778**	GO:0012505	C:endomembrane system	0,95			2-22;303-331;457-475	2-22	303-331	457-475	
**FBgn0036910**	GO:0012505	C:endomembrane system	0,93			2-20;296-324;452-470	2-20	296-324	452-470	
**FBgn0037601**	GO:0012505	C:endomembrane system	0,94			2-21;294-322;450-469	2-21	294-322	450-469	
**FBgn0038005**	GO:0012505	C:endomembrane system	0,95			2-21;279-307;435-453	2-21	279-307	435-453	
**FBgn0038006**	GO:0012505	C:endomembrane system	0,95			2-20;284-312;440-458	2-20	284-312	440-458	
**FBgn0038007**	GO:0012505	C:endomembrane system	0,95			2-21;284-312;440-458	2-21	284-312	440-458	
**FBgn0038076**	GO:0012505	C:endomembrane system	0,95			2-21;286-314;442-460	2-21	286-314	442-460	
**FBgn0038095**	GO:0012505	C:endomembrane system	0,93			2-20;302-330;456-474	2-20	302-330	456-474	
**FBgn0038194**	GO:0012505	C:endomembrane system	0,95			2-21;301-329;455-473	2-21	301-329	455-473	
**FBgn0038236**	GO:0012505	C:endomembrane system	0,95			2-21;284-312;440-458	2-21	284-312	440-458	
**FBgn0039006**	GO:0012505	C:endomembrane system	0,92			2-22;305-333;459-477	2-22	305-333	459-477	
**FBgn0086917**	GO:0012505	C:endomembrane system	0,94			6-38;309-336;501-520	6-38	309-336	501-520	
